# RETRACTED: Al-Salmi, F.A.; Hamza, R.Z. Efficacy of Vanadyl Sulfate and Selenium Tetrachloride as Anti-Diabetic Agents against Hyperglycemia and Oxidative Stress Induced by Diabetes Mellitus in Male Rats. *Curr. Issues Mol. Biol.* 2022, *44*, 94–104

**DOI:** 10.3390/cimb46060331

**Published:** 2024-05-31

**Authors:** Fawziah A. Al-Salmi, Reham Z. Hamza

**Affiliations:** Biology Department, College of Sciences, Taif University, P.O. Box 11099, Taif 21944, Saudi Arabia; f.alsalmi@tu.edu.sa

*Current Issues in Molecular Biology* Editorial Office retracts the article titled “Efficacy of Vanadyl Sulfate and Selenium Tetrachloride as Anti-Diabetic Agents against Hyperglycemia and Oxidative Stress Induced by Diabetes Mellitus in Male Rats” [1].

Following publication, concerns were brought to the attention of the Editorial Office regarding an overlap of images between different figures in the article [1] and between article [1] and other previously published article [2].

Adhering to our complaint procedure, an investigation was conducted by the Editorial Office and Editorial Board that confirmed an overlap between:

- Figure 2C [1] with Figure 2D [1],
- Figure 2A [1] with Figure 3A [2],
- Figure 2C [1] with Figure 3D [2],
- Figure 2D [1] with Figure 3D [2],
- Figure 2B [1] with Figure 3B [2],
- Figure 3B [1] with Figure 4B [2].

While the authors fully cooperated with the investigation, they were unable to satisfactorily explain the overlapping panels within the above-mentioned figures, nor could they provide raw images of sufficient high resolution and quality to validate these findings. As a result, the Editorial Office and Editorial Board have lost confidence in the reliability of these findings and have decided to retract this article [1] as per MDPI’s retraction policy (https://www.mdpi.com/ethics#_bookmark30 accessed on 6 May 2024) and in line with the Committee on Publication Ethics retraction guidelines (https://publicationethics.org/retraction-guidelines accessed on 6 May 2024).

This retraction was approved by the Editor-in-Chief of the journal *Current Issues in Molecular Biology*.

The authors did not agree to this retraction. 
